# The Capacity to Produce Hydrogen Sulfide (H_2_S) via Cysteine Degradation Is Ubiquitous in the Human Gut Microbiome

**DOI:** 10.3389/fmicb.2021.705583

**Published:** 2021-10-20

**Authors:** Domenick J. Braccia, Xiaofang Jiang, Mihai Pop, A. Brantley Hall

**Affiliations:** ^1^Center for Bioinformatics and Computational Biology, University of Maryland, College Park, College Park, MD, United States; ^2^National Library of Medicine, National Institutes of Health, Bethesda, MD, United States; ^3^Department of Computer Science, University of Maryland, College Park, College Park, MD, United States; ^4^Department of Cell Biology and Molecular Genetics, University of Maryland, College Park, College Park, MD, United States

**Keywords:** metagenomics, hydrogen sulfide, human health, inflammatory bowel disease (IBD), colorectal cancer, microbiome, gut microbiome

## Abstract

As one of the three mammalian gasotransmitters, hydrogen sulfide (H_2_S) plays a major role in maintaining physiological homeostasis. Endogenously produced H_2_S plays numerous beneficial roles including mediating vasodilation and conferring neuroprotection. Due to its high membrane permeability, exogenously produced H_2_S originating from the gut microbiota can also influence human physiology and is implicated in reducing intestinal mucosal integrity and potentiating genotoxicity and is therefore a potential target for therapeutic interventions. Gut microbial H_2_S production is often attributed to dissimilatory sulfate reducers such as *Desulfovibrio* and *Bilophila* species. However, an alternative source for H_2_S production, cysteine degradation, is present in some gut microbes, but the genes responsible for cysteine degradation have not been systematically annotated in all known gut microbes. We classify mechanisms of cysteine degradation into primary, secondary, and erroneous levels of H_2_S production and perform a comprehensive search for primary, secondary, and erroneous cysteine-degrading enzymes in 4,644 non-redundant bacterial genomes from the Unified Human Gastrointestinal Genome (UHGG) catalog. Of the 4,644 genomes we have putatively identified 2,046 primary, 1,951 secondary, and 5 erroneous cysteine-degrading species. We identified the presence of at least one putative cysteine-degrading bacteria in metagenomic data of 100% of 6,623 healthy subjects and the expression of cysteine-degrading genes in metatranscriptomic data of 100% of 736 samples taken from 318 individuals. Additionally, putative cysteine-degrading bacteria are more abundant than sulfate-reducing bacteria across healthy controls, IBD patients and CRC patients (*p* < 2.2e-16, Wilcoxon rank sum test). Although we have linked many taxa with the potential for cysteine degradation, experimental validation is required to establish the H_2_S production potential of the gut microbiome. Overall, this study improves our understanding of the capacity for H_2_S production by the human gut microbiome and may help to inform interventions to therapeutically modulate gut microbial H_2_S production.

## Introduction

**FIGURE 1:**
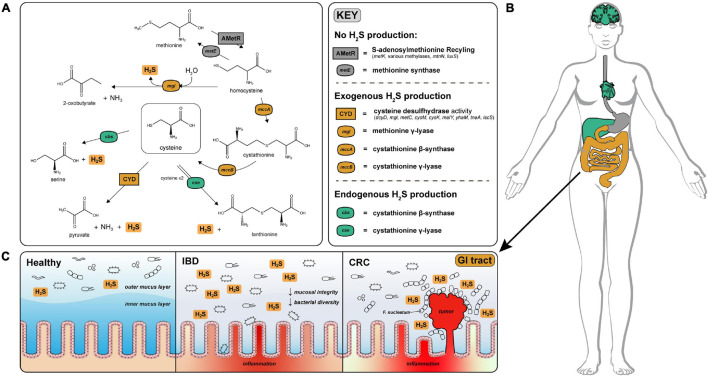
H_2_S production via cysteine degradation in the human gut microbiome. **(A)** Pathways of H_2_S production via cysteine degradation in the human gut microbiome. Pathways with labels ending in “activity” refer to a set of genes that convert cysteine to the indicated products. Cysteine desulfurase activity (CYD) = (*dcyD, mgl, metC, cysM, cysK, malY, yhaM, tnaA, iscS*). AMetR = AdoMet recycling present in *Bacillus subtilis* (*metK*, various methylases, *mtnN*, *luxS*) (Hullo et al., 2007). **(B)** Visualization of H_2_S production across human tissues (image obtained from Papatheodorou et al., 2020). H_2_S is produced endogenously in the brain, liver, and heart via cystathionine gamma-lyase and cystathionine beta-synthase and is tightly regulated to avoid toxic effects of H_2_S overproduction. Color coded organs refer to the type of H_2_S production active in those organs. **(C)** Physiological effects of H_2_S on the healthy, IBD and CRC gut. H_2_S contributes to the degradation of the protective mucosal barrier which could cause or exacerbate inflammation and infection by opportunistic species for patients diagnosed with IBD. In CRC, *Fusobacterium nucleatum* is closely associated with colonic tumors and are well known H_2_S producers (Castellarin et al., 2012).

Hydrogen sulfide (H_2_S) is a consequential molecule produced by the gut microbiota with pleiotropic effects on human physiology. It is one of the three physiological gasotransmitters, along with carbon monoxide and nitric oxide, and is produced endogenously in many tissues including, but not limited to, the brain, heart and liver (Wang, 2009). Endogenous H_2_S production occurs via the enzymes cystathionine beta-synthase (*cbs*), cystathionine gamma-lyase (*cse*) and 3-mercaptopyruvate sulfur transferase (*3-mst*) (Kimura, 2009). *cbs*, *cse* and *3-mst* are tightly regulated pyridoxal-5′-phosphate (PLP)-dependent enzymes and produce H_2_S primarily from the degradation of cysteine (Percudani and Peracchi, 2003; Figure 1B). H_2_S produced by these enzymes plays a litany of physiological roles including: suppression of oxidative stress in the brain, regulation of blood pressure through vasodilation and protection of hepatic stellate cells from cirrhosis in the liver (Wang, 2012). As a result, abnormally low endogenous levels of H_2_S are hypothesized to be an underlying cause of peripheral artery disease, and efforts have been made to measure serum levels of H_2_S quickly and non-invasively as a proxy for early detection of peripheral artery disease (Shekarriz et al., 2020).

Microbes in the gastrointestinal tract also contribute to H_2_S production in humans. A majority of the microbially produced H_2_S originates in the colon, where estimates of luminal concentrations of H_2_S range from 0.3 to 3.4 mM (Suarez et al., 1997). The serum concentration of H_2_S in healthy individuals is difficult to measure but is estimated to range from 34.0 to 36.4 μM (Furne et al., 2008). H_2_S readily diffuses across the intestinal epithelium and can enter circulation influencing host physiology (Furne et al., 2001). Excessive production of H_2_S by gut microbes has been linked with decreased mucosal integrity through reduction of mucosal disulfide bonds (Blachier et al., 2021), inhibition of colonocyte butyrate oxidation via cytochrome-c inhibition (Gibson et al., 1988), and genotoxicity (Furne et al., 2001; Figure 1C). A prime example of the gut microbiome effecting gut health is association of *Fusobacterium nucleatum*—a known H_2_S producer—with colonic tumors (Castellarin et al., 2012; Figure 1C).

While the mammalian pathways of H_2_S production have been well studied, the contribution of gut-microbial H_2_S production to circulating H_2_S levels and the subsequent systemic effects on human physiology are largely unknown. The first step toward a better understanding of the effects of H_2_S on human physiology is to identify which microbial species are responsible for H_2_S production. There are two major sources for H_2_S production in the human gut microbiota, dissimilatory sulfate reduction (DSR) and the degradation of the sulfur-containing amino acids cysteine and methionine (Carbonero et al., 2012). We must note that sulfate is first reduced to sulfite before H_2_S is produced, however, we refer to this process as sulfate reduction for the remainder of this work.

In the literature, H_2_S production is often attributed to the well-characterized dissimilatory sulfate reduction pathway (Wang, 2012). Common representatives of sulfate-reducing bacteria (SRB) are found in the phylum *Desulfobacterota* (recently reclassified from the class *Deltaproteobacteria*) with *Desulfovibrio* spp. and *Bilophila wadsworthia* being the most abundant representatives in the human gut (Gibson et al., 1988; Waite et al., 2020). Sulfate and sulfite are used by SRB as terminal electron acceptors for anaerobic respiration (Levine et al., 1998). While SRB are prevalent in human populations, their relative abundances are generally very low and are dependent on ecological interactions with other hydrogenotrophs, such as methanogens and acetogens (Gibson et al., 1988; Deng et al., 2015; Yao et al., 2018). Methane (CH_4_) is primarily produced by the methanogen *Methanobrevibacter smithii* (Miller et al., 1982) and is one of the primary gases present in mammalian flatus. Sulfate-reducing bacteria and methanogens have been historically considered mutually exclusive in microbial communities due to the competition for hydrogen (Gibson et al., 1988). However, experiments carried out on human flatus have shown that both H_2_S and CH_4_ production occurs simultaneously in some individuals, seemingly contradicting the notion that methanogens and sulfate-reducing bacteria cannot co-exist (Suarez et al., 1997).

Unlike the comprehensively characterized pathways for dissimilatory sulfate reduction, the species of the gut microbiome responsible for H_2_S production via degradation of sulfur-containing amino acids (cysteine and methionine) have not been comprehensively characterized. Gut microbial involvement in amino acid fermentation has garnered recent attention, as many physiologically relevant downstream metabolites are produced by gut microbial degradation of amino acids (Lin et al., 2017; Figure 1A). Depending on dietary intake, a pool of sulfur-containing amino acids is available for fermentation by gut microbiota (Silvester and Cummings, 1995). Various studies have demonstrated that cysteine supplementation leads to far more H_2_S production than inorganic sulfate supplementation underscoring the comparative importance of the cysteine-degradation pathway in total H_2_S production (Levine et al., 1998; Deng et al., 2015; Yao et al., 2018).

It is important to delineate between H_2_S produced via dissimilatory sulfate reduction and H_2_S produced via cysteine degradation because different approaches are necessary to modulate these two sources of H_2_S production. Because of the poor annotation of the genes which produce H_2_S via cysteine degradation across species of the gut microbiome, the relative contributions of cysteine-degradation and sulfate-reduction to overall exogenous H_2_S production are unclear. To address this gap, we designed a bioinformatic approach to first identify putative cysteine-degrading bacteria in the human gut microbiome and then compared the relative abundances of putative cysteine-degrading bacteria and sulfate-reducing bacteria across metagenomic data from Inflammatory Bowel Disease (IBD), colorectal cancer (CRC), and healthy cohorts (Supplementary Figure 1).

## Results

To identify species capable of H_2_S production via cysteine-degradation in the human gut microbiome, we curated profile Hidden Markov Models (pHMMs) of enzymes with experimental evidence of H_2_S production via cysteine-degradation and classified them into primary, secondary and erroneous producers of H_2_S. Enzymes which produce H_2_S via cysteine degradation as their primary function are labeled “primary” (*dcyD*, *yhaM*, *mgl*, *sseA*) and enzymes which also participate in separate mechanism(s), such as the transsulfuration pathway and maltose regulon modulation, have been labeled “secondary” (*metC*, *malY*, *cysK*, *cysM*, *mccB*). Enzymes which have a well-defined function other than H_2_S production via cysteine-degradation are labeled “erroneous” (*tnaA*, *iscS*, *mccA*). Please see Supplementary Note 1 for more information.

### Cysteine-Degrading Genes Are Widely Distributed in the Human Gut Microbiome

**FIGURE 2:**
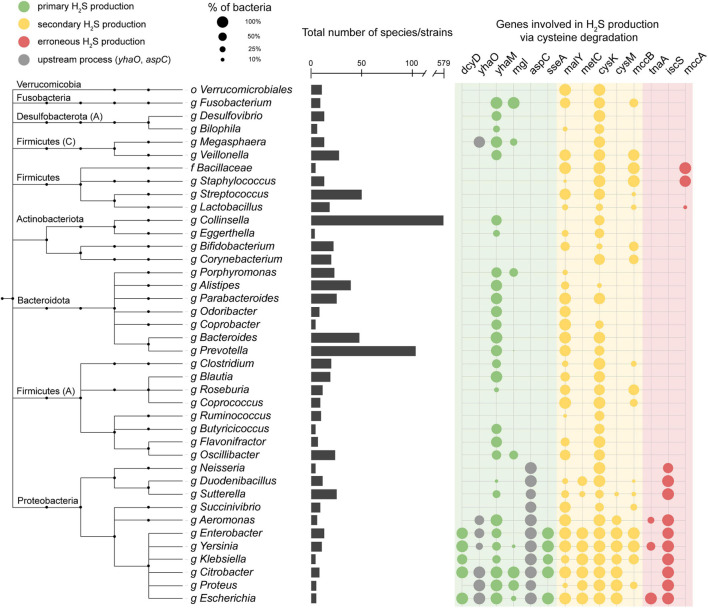
Presence of cysteine-degrading genes across the human gut microbiome. Important genera of the human gut microbiome and the presence/absence of cysteine-degrading genes in each clade. The taxonomic tree is obtained from the Unified Human Gastrointestinal Genome collection (UHGG) (Almeida et al., 2020) which is built on the Genome Taxonomy Database (GTDB) (Chaumeil et al., 2020). Phyla names are annotated on the left side. Phyla followed by a capital letter, e.g., Desulfobacterota (A), indicate a novel phyla classified by the GTDB-tk (Chaumeil et al., 2020). The bar chart in the center of the figure represents the number of species contained under each genus or higher clade. The color of the circles on the right indicates whether the gene is a primary (green), secondary (yellow) or erroneous (red) producer of H_2_S (Supplementary Note 1). The circles on the right side represent the number of species in each clade that contain hits to the genes specified. Nodes collapsed at levels higher than genus are because all genomes in that clade contain the same combination of genes reported in the grid on the right. The full, untruncated version of this figure is available in the supplementary information (Supplementary Figure 2).

We performed a homolog search for these H_2_S producing enzymes across 4,644 species in the Unified Human Gastrointestinal Genome (UHGG) collection (Almeida et al., 2020) using HMMER (2021) (Figure 2 and Supplementary Figure 1A). This collection comprises 4,644 non-redundant genome sequences from species representatives generated by clustering 204,938 genome sequences from bacteria known to inhabit the human gut. Of the representative UHGG species, 44.1% (2,046/4,644) contain one or more primary cysteine-degrading gene, 42.0% (1,951/4,644) contain one or more secondary cysteine-degrading gene, and 1.1% (5/4,644) contain one or more erroneous cysteine-degrading gene. Aside from known cysteine-degrading bacterial species compiled in the manual curation step, an additional 3,065 species from the UHGG catalog were found to contain one or more cysteine-degrading genes (Figure 2, Supplementary Figure 2, and Supplementary Table 2).

**FIGURE 3:**
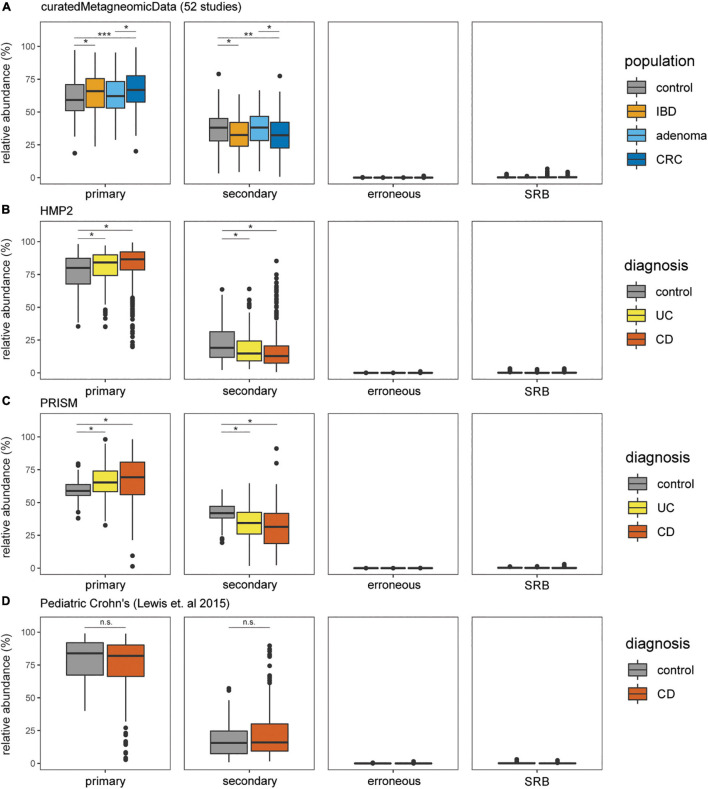
Putative primary and secondary cysteine-degrading bacteria are more prevalent than (SRB) among individuals with IBD, CRC, and healthy controls. Relative abundances of putative cysteine-degrading bacteria and sulfate-reducing bacteria across healthy and diseased populations. Relative abundances were calculated using Kraken2 (Wood et al., 2019) (see section “Materials and Methods”). **(A)** Data obtained from curatedMetagenomicData (Pasolli et al., 2017). Number of samples per disease category: control = 560, CRC = 352, adenoma = 143, IBD = 148. **(B)** Data obtained from HMP2 (Almeida et al., 2020). Number of samples per disease category: non-IBD = 359, ulcerative colitis (UC) = 367, Crohn’s disease (CD) = 591. **(C)** Data obtained from PRISM (Franzosa et al., 2019). Number of samples per disease category: control = 56, UC = 76, CD = 88. **(D)** Data obtained from study on Pediatric Crohn’s Disease (Lewis et al., 2015). Number of samples per disease category: control = 26, CD = 86.

The prevalence and relative abundance of putative cysteine-degrading bacteria and sulfate-reducing bacteria was calculated for 10,700 metagenomic samples from healthy, IBD, CRC, and adenoma cohorts (Lewis et al., 2015; Pasolli et al., 2017; Franzosa et al., 2019; Proctor and Huttenhower, 2019). Among the 6,632 healthy subjects, there is a markedly higher relative abundance of putative primary and secondary cysteine-degrading bacteria compared to sulfate-reducing bacteria (*p* < 2.2e-16, two-sided Wilcoxon Rank Sum Test) (Figure 3). This suggests that cysteine-degradation may contribute considerably to H_2_S production for the average healthy person. Cysteine-degrading genes are also widespread in healthy populations with 100% of the 6,623 healthy subjects containing at least one putative cysteine-degrading bacteria in their gut microbiome (Figure 3).

### Increased Relative Abundance of Putative Hydrogen Sulfide Producing Bacteria in the Inflammatory Bowel Disease and Colorectal Cancer Gut Microbiomes

We assessed the relative abundance of putative cysteine-degrading bacteria and sulfate-reducing bacteria in individuals with the two most common clinical manifestations of inflammatory bowel disease (IBD), Crohn’s disease and ulcerative colitis, colorectal cancer (CRC) and healthy controls (Schirmer et al., 2019). Putative primary and secondary cysteine-degrading bacteria are significantly more abundant than sulfate-reducing bacteria across IBD and CRC populations from metagenomic samples obtained from curatedMetagenomicData (Pasolli et al., 2017), the Integrative Human Microbiome Project 2 (HMP2) (Proctor and Huttenhower, 2019), PRISM (Lewis et al., 2015; Franzosa et al., 2019) (all *p* < 2.2 × 10^–16^) (Figures 3A–D).

Putative primary cysteine-degrading bacteria are significantly more abundant in CRC than in the control groups (W = 123,784, *p* = 7.4 × 10^–11^) (Figure 3A) while putative secondary cysteine-degrading bacteria were found to be significantly less abundant in CRC compared to healthy controls (W = 79,734; *p* = 1.2 × 10^–6^). A similar trend follows for ulcerative colitis and Crohn’s disease compared to healthy controls. Putative primary cysteine-degrading bacteria are significantly more abundant in IBD when compared to healthy controls and putative secondary cysteine-degrading bacteria are significantly less abundant than in healthy controls (Figures 3B,C). Within pediatric Crohn’s disease, there is no apparent difference in the abundance of putative primary and secondary cysteine-degrading bacteria (Figure 3D).

### Primary and Secondary Cysteine-Degrading Genes Are Actively Expressed in the Human Gut Microbiome

**FIGURE 4:**
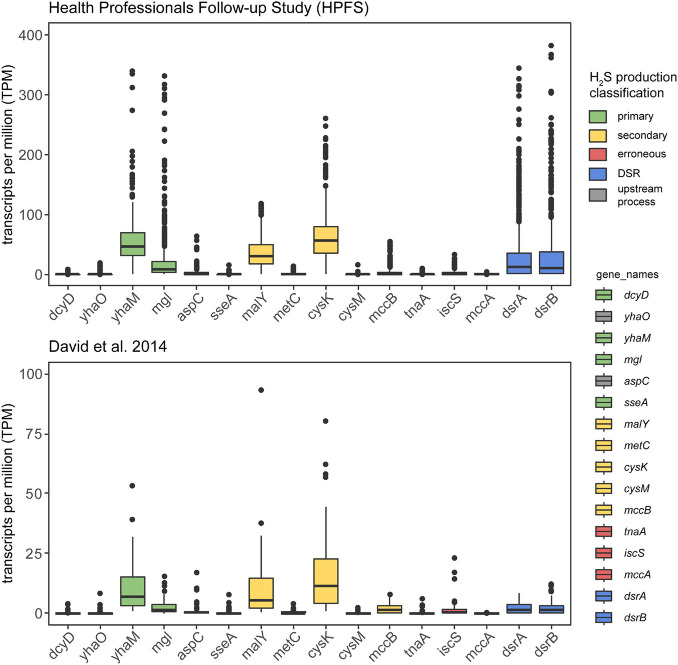
Cysteine-degrading genes are actively expressed in healthy adults. This analysis confirms that the H_2_S producing genes considered in this work are actively expressed in healthy adults. The y-axis displays TPM counts for each gene involved in H_2_S production via both cysteine degradation and dissimilatory sulfate reduction. The legend on the right indicates the mode of H_2_S production for each of the genes examined. Metatranscriptomic reads from HPFS (number of individuals = 308, number of samples = 677) (David et al., 2014; Abu-Ali et al., 2018) (number of individuals = 10, number of samples = 59) (David et al., 2014) were aligned to gene hit sequences identified in the homolog search step using salmon (Patro et al., 2017) and visualized by the ggplot2 (Wickham, 2011) package in R (Supplementary Figure 1C). Certain primary and secondary H_2_S producing genes are actively produced by gut bacteria in healthy adults and erroneous producers of H_2_S appear to be less actively transcribed.

To confirm *in vivo* transcription of cysteine-degrading genes and sulfate-reducing genes in the human gut microbiome, we analyzed 736 metatrasncriptomic sequencing runs from 318 individuals across two studies: (1) The Health Professionals Follow-up Study (number of individuals = 308, number of samples = 677) (Abu-Ali et al., 2018) and (2) David et al. (2014) (number of individuals = 10, number of samples = 59). Both studies took multiple samples from participants over the course of time and/or dietary intervention, hence the distinction between number of individuals and number of samples. Our analysis revealed that 86.5% (637/736) of samples show expression of at least one primary cysteine-degrading gene, 89.7% (660/736) of samples showed expression of at least one secondary cysteine-degrading gene and 84.1% (619/736) of samples showed expression of dissimilatory sulfate reduction genes *dsrA* and *dsrB* (Figure 4 and Supplementary Figure 4). *yhaM* and *mgl* are the most actively transcribed primary cysteine-degrading genes with *malY* and *cysK* being the most actively transcribed secondary cysteine-degrading genes. The erroneous cysteine-degrading genes *tnaA*, *iscS*, and *mccA* are considerably less transcribed across healthy human gut microbiomes (Figure 4). These results suggest that primary and secondary cysteine degradation could be prominent pathways of H_2_S production in some individuals.

### Core Dissimilatory Sulfate Reduction Genes and Methanogenesis Genes Are Co-expressed *in vivo*

Previously, *in vitro* assays have indicated that methanogens and sulfate-reducing bacteria compete for hydrogen and may thus mutually exclude one another (Gibson et al., 1988). However, through analysis of 736 metatranscriptomic samples obtained from 318 individuals across two studies (David et al., 2014; Abu-Ali et al., 2018), we observed that core genes involved in dissimilatory sulfate reduction and methanogenesis are simultaneously expressed in 25.8% (175/677) of samples from the HPFS study and in 11.9% (7/59) of samples from the David et al. (2014) study (Supplementary Figure 4). This suggests that the mutual exclusivity of sulfate reducing bacteria and methane producing bacteria observed *in vitro* does not necessarily apply to the complex biogeography of the gut.

## Discussion

Due to its role as a mammalian gasotransmitter, H_2_S plays important roles in maintaining physiological homeostasis. However, H_2_S may also cause deleterious effects in a concentration-dependent manner. Therefore, it is of great importance to understand the sources of exogenous H_2_S production in the gut in order to tease out the links between H_2_S and human physiology. The source of gut microbial H_2_S production is often attributed to dissimilatory sulfate reduction, with far less attention given to H_2_S production via the degradation of the sulfur-containing amino acid cysteine. In fact, there has not been a microbiome-wide annotation of the potential for H_2_S production via cysteine degradation. The systematic annotation we performed in this study expands our understanding of which species can potentially produce H_2_S in the gut, the majority of which have not been previously reported to have the capability for H_2_S production. Our analysis of shotgun sequenced metagenomic data from 10,700 metagenomic samples revealed that putative cysteine-degrading bacteria are ubiquitous inhabitants of the human gut microbiome and are present at significantly higher relative abundance than sulfate-reducing bacteria. Furthermore, our analysis of 736 metatranscriptomic samples from 318 healthy individuals demonstrates that primary and secondary cysteine-degrading genes are, in fact, actively expressed in the gut. These results suggest that primary and secondary cysteine degradation could be prominent pathways of H_2_S production in some individuals. Therefore, cysteine degradation is an important aspect to consider when designing studies to assess the effects of H_2_S on human health or modulate gut microbial H_2_S production.

We also explored the relative abundance of putative cysteine-degrading bacteria in IBD and CRC to understand whether these bacteria could contribute to or promote disease progression. We found that primary putative cysteine-degrading bacteria are significantly more abundant in CRC samples than in healthy controls. While relative abundances of sulfate-reducing bacteria are modestly higher in CRC compared to healthy controls, primary putative cysteine-degrading bacteria are far more abundant. This finding corroborates previous studies linking H_2_S and the progression of CRC (Castellarin et al., 2012) and highlights the need to identify the dominant source of H_2_S in the CRC gut. Importantly, it remains to be elucidated whether or not this difference in relative abundance translates to higher production of H_2_S via cysteine degradation.

Prior studies suggested that methanogens and sulfate-reducing bacteria are mutually exclusive, potentially due to their competition for hydrogen. However, subsequent studies have reported the presence of both CH_4_ and H_2_S in the human flatus (Suarez et al., 1997), seemingly contradicting this notion of mutual exclusivity of CH_4_ and H_2_S producing bacteria. To resolve this discrepancy, we examined the transcriptional co-occurrence of methanogens and sulfate-reducing bacteria, and cysteine-degrading bacteria in the human gut and found the co-occurrence of all three pathways. This discrepancy between *in vitro* experiments and *in vivo* observations could be explained by the complex biogeography of the gut in which methanogens and sulfate-reducing bacteria occupy distinct niches or from H_2_S production via cysteine degradation.

The primary limitation of this study is the lack of experimental confirmation of primary, secondary and erroneous putative cysteine-degrading bacteria. This could be addressed by performing a screen for H_2_S production via cysteine degradation for all culturable strains that we have identified as putative cysteine-degrading bacteria. There are many reactions in which H_2_S is formed as an intermediate, such as assimilatory sulfate reduction, however, these reactions do not result in significant production of H_2_S and are thus not relevant to total H_2_S production by the gut microbiome. Therefore, we limited our search for H_2_S producing bacteria to pathways in which H_2_S was the endpoint, or byproduct, and not just an intermediate of the pathway. Our search identified the genes for dissimilatory sulfate reduction in *Eggerthella* and *Gordonibacter* species. We have included these species as sulfate-reducing bacteria though there is little evidence to suggest that these species are true sulfate reducers (Müller et al., 2015; Nguyen et al., 2018). Experimental validation of these claims is necessary to confirm *Eggerethella* spp. and *Gordonibacter* spp. as non-sulfate-reducing bacteria. We also note that our search for H_2_S producing genes included only the 4,644 representative genomes in UHGG. The full UHGG collection contains 204,938 non-redundant genomes with core and accessory gene information that may contain other putative H_2_S-producing sub-species that we did not analyze. Another potential shortcoming of this analysis is the overrepresentation of western countries in the data pool used. An expanded set of samples would be required to claim that primary and secondary putative cysteine-degrading bacteria are globally prevalent in the human gut microbiome. Finally, we note that sulfate-reducing bacteria may be mucosally associated and present at low relative abundances which could mean that stool metagenomics may underestimate the true abundance of sulfate-reducing bacteria in the human gut.

In conclusion, we show that the relative abundance of primary putative cysteine-degrading bacteria is significantly higher than sulfate-reducing bacteria across healthy individuals as well as individuals with colorectal cancer and inflammatory bowel disease. These results bolster previous studies suggesting the importance of dietary cysteine in gut microbial H_2_S production. The systematic annotation of putative H_2_S-producing species performed in this study can serve as a resource for future studies examining the links between H_2_S and disease and could help these studies to tease out the concentration-dependent effects of H_2_S on human health. Overall, this work informs future approaches to modulate gut microbial H_2_S production via dietary interventions and may lead to an improved understanding of the complex interplay between H_2_S and human health and disease.

## Materials and Methods

Supplementary Figure 1 provides a visual overview of the computational workflow carried out in this manuscript. This workflow is available at https://github.com/dombraccia/H2S.

### Curation of Profile Hidden Markov Models Involved in Cysteine-Degradation and Sulfate-Reduction

We performed a literature search to identify genes involved in H_2_S production via dissimilatory sulfate reduction and sulfur-containing amino acid degradation by gut bacteria (Awano et al., 2005; Shatalin et al., 2011; Suwabe et al., 2011; Carbonero et al., 2012; Nava et al., 2012; Shimada et al., 2016).

The pHMMs corresponding to the gene families responsible for H_2_S production were obtained from TIGRFAM or HAMAP (the pHMMs used are listed in Supplementary Table 1). If neither a TIGRFAM nor HAMAP pHMM was available for a given gene *or* the profile was too broadly defined, we opted to construct a custom pHMM to represent the gene of interest. Custom pHMMs were constructed by 1. concatenating amino acid sequences pertaining to the gene of interest 2. performing a multiple sequence alignment over these amino acid sequences using MUSCLE (Edgar, 2004) 3. constructing the pHMMs from the multiple sequence alignments using hmmbuild from the HMMER tool suite (HMMER, 2021). This was done to avoid identifying spurious homolog hits in the subsequent search step. pHMMs are detailed in Supplementary Table 1.

### Search for Putative Hydrogen Sulfide Producing Bacteria in the Human Gut

The pHMMs of H_2_S producing genes were searched against 4,644 genome sequences from UHGG (Almeida et al., 2020) using the hmmscan method from hmmer v3.1 (HMMER, 2021) (Supplementary Figure 1A). Hits were filtered based on a conservative *E*-value threshold (*E*-value < 1 × 10^–110^) and an additional bit score threshold was applied for TIGRFAM pHMMs to avoid calling spurious hits. Hits to HAMAP and custom pHMMs were only filtered based on the *E*-value threshold. Next, the bacterial genomes receiving hits were categorized into putative primary, secondary, and erroneous cysteine degraders based on the known mechanisms of the genes used in the search space. Genomes receiving hits to the *dsrAB* operon were labeled as sulfate-reducing bacteria. Please see Supplementary Note 1 for a detailed description of the primary, secondary, and erroneous classification scheme. Primary, secondary, and erroneous putative cysteine-degrading bacteria across UHGG were then visualized by uploading a taxonomic tree in newick tree format to the iTOL (Letunic and Bork, 2019) web interface (Figure 2). Gene containments for each of the UHGG genomes were converted to the EXTERNALSHAPE file format specified by iTOL documentation^1^ and uploaded to the iTOL visualization file from the previous step.

### Calculating Relative Abundances With Kraken 2

Raw sequencing reads for samples from curatedMetagenomicData, HMP2, PRISM, and Lewis et al. (2015) were downloaded and extracted with NCBI’s SRA toolkit v2.10.9 (SRA-Tools, 2021). Quality control and adapter trimming of the fastq sequence files were done with the Trim Galore wrapper v0.6.6 (Babraham Bioinformatics-Trim Galore, 2021). To remove potential human contaminants, quality-trimmed reads were screened against the human genome (hg19) with Bowtie2 v2.4.2 (Langmead and Salzberg, 2012). Taxonomy profiling of the cleaned metagenomic reads were generated using Kraken 2 (2.0.8-beta) (Wood et al., 2019) to estimate the relative abundance of bacterial species present in each dataset. These relative abundances are then processed and plotted in Figure 3.

### Transcriptomic Analysis of Hydrogen Sulfide Producing Genes and Methane Producing Genes

We sought to confirm the active expression of H_2_S producing genes and CH_4_ producing genes alongside the existing genomic evidence presented using metatranscriptomic data from David et al. (2014) and the Health Professionals Follow-up Study (Abu-Ali et al., 2018). Confirming the expression of H_2_S producing and CH_4_ producing genes involved the following steps: 1. Metadata for samples was downloaded from the SRA run selector.^2^ 2. Raw sequencing data was downloaded using fasterq-dump from the SRA toolkit version 2.10.9 (SRA-Tools, 2021). 3. Manually curated H_2_S producing genes and CH_4_ producing genes were given as input to salmon index (Patro et al., 2017). 4. Raw RNA-seq data were then quantified against the manually curated gene sequence database using the salmon quant command with the –validateMappings option on for better performance. The raw counts of reads mapped per gene were normalized to TPM values for downstream analysis. The threshold for considering an H_2_S gene “expressed” was TPM > = 10. A sample was said to be “methane producing” if ≥80% of the 16 genes involved in the methanogenesis pathway recruited one or more read mapping. These genes are listed in the *x*-axis of Supplementary Figure 3. The results were then parsed with custom shell and R scripts and visualized in Figure 4 and Supplementary Figure 3 using the R package ggplot2 (Wickham, 2011). All scripts and workflow is available at https://github.com/dombraccia/H2S.

## Data Availability Statement

Publicly available datasets were analyzed in this study. These data can be found here: HMP2: https://portal.hmpdacc.org/; PRISM: available via SRA with BioProject number PRJNA400072; Lewis et al. (2015): available via SRA under SRP057027; UHGG: https://www.ebi.ac.uk/ena/data/view/PRJEB33885; David et al. (2014): available via SRA with BioProject number PRJNA202303; HPFS: available via SRA with BioProject number PRJNA354235; and curatedMetagenomicData: available via R/Bioconductor by running ‘BiocManager::install(“curatedMetagenomicData”)’ in an R console.

## Author Contributions

DB performed all analyses. AH, XJ, and MP conceived and supervised the work. All authors contributed to manuscript preparation.

## Conflict of Interest

The authors declare that the research was conducted in the absence of any commercial or financial relationships that could be construed as a potential conflict of interest.

## Publisher’s Note

All claims expressed in this article are solely those of the authors and do not necessarily represent those of their affiliated organizations, or those of the publisher, the editors and the reviewers. Any product that may be evaluated in this article, or claim that may be made by its manufacturer, is not guaranteed or endorsed by the publisher.

## Footnotes

1
https://itol.embl.de/help.cgi

2
https://trace-ncbi-nlm-nih-gov

## Supplementary Material

Supplementary Figure 1 Computational workflow. **(A)** Representative bacterial genomes from the United Human Gastrointestinal Genomes collection (UHGG) (Almeida et al., 2020) were downloaded and profile hidden markov models (pHMMs) representing cysteine-degrading genes were manually curated after extensive literature search (Supplementary Note 1 and Supplementary Table 1). pHMMs were searched across the UHGG database using hmmscan (HMMER) (HMMER, 2021) and gene containments are reported in Figure 2 and Supplementary Figure 2. **(B)** Relative abundances of putative primary, secondary and erroneous cysteine-degrading bacteria were compared across healthy, IBD and CRC individuals and reported in Figure 3. **(C)** Metatranscriptomic sequencing reads from HPFS (number of individuals = 308, number of samples = 677) (David et al., 2014; Abu-Ali et al., 2018) (number of individuals = 10, number of samples = 59) (David et al., 2014) were aligned and quantified against UHGG gene hits using salmon (Patro et al., 2017) and results are visualized in Figure 4 using ggplot2 (Wickham, 2011).

Supplementary Figure 2 Prevalence of cysteine-degrading genes across UHGG. Depiction of complete set of cysteine-degrading genes present across the UHGG collection. All genera from Figure 2 are included here as well as less prominent bacteria and computationally assembled genomes. Left: complete taxonomic tree of UHGG genomes. Middle: bar chart representing the total number of genomes under specified clade. Right: presence/absence of putative primary, secondary and erroneous cysteine-degrading genes among the UHGG representative genome collection.

Supplementary Figure 3 Gene expression of methane producing genes in the human gut. Genes involved in the production of CH_4_ by *Methanobrevibacter smithii* expressed in healthy individuals. Metatranscriptomic reads from the Health Professionals Follow-up Study (HPFS) (number of individuals = 308, number of samples = 677) (David et al., 2014; Abu-Ali et al., 2018) (number of individuals = 10, number of samples = 59) (David et al., 2014) were aligned to genes involved in methane production by *Methanobrevibacter smithii* using salmon (Patro et al., 2017) and visualized by R package ggplot2 (Wickham, 2011; Supplementary Figure 1).

Supplementary Figure 4 Co-expression of DSR, methanogenesis and cysteine-degrading genes by the human gut microbiome. Genes involved in the production of CH_4_ via methanogenesis and H_2_S via dissimilatory sulfate reduction (DSR) and cysteine-degradation expressed in healthy individuals. Metatranscriptomic reads from the Health Professionals Follow-up Study (HPFS) (Abu-Ali et al., 2018) (number of individuals = 308, number of samples = 677) and (David et al., 2014; Abu-Ali et al., 2018) (number of individuals = 10, number of samples = 59) were aligned and quantified with salmon (Patro et al., 2017) and visualized by R package ggplot2 (Wickham, 2011).
