# Supplementary figures and images for: The Capacity to Produce Hydrogen Sulfide (H2S) via Cysteine Degradation Is Ubiquitous in the Human Gut Microbiome
Source: Front Microbiol. 2021 Oct 20;12:705583. doi: 10.3389/fmicb.2021.705583 (PMC8564485; doi:10.3389/fmicb.2021.705583)

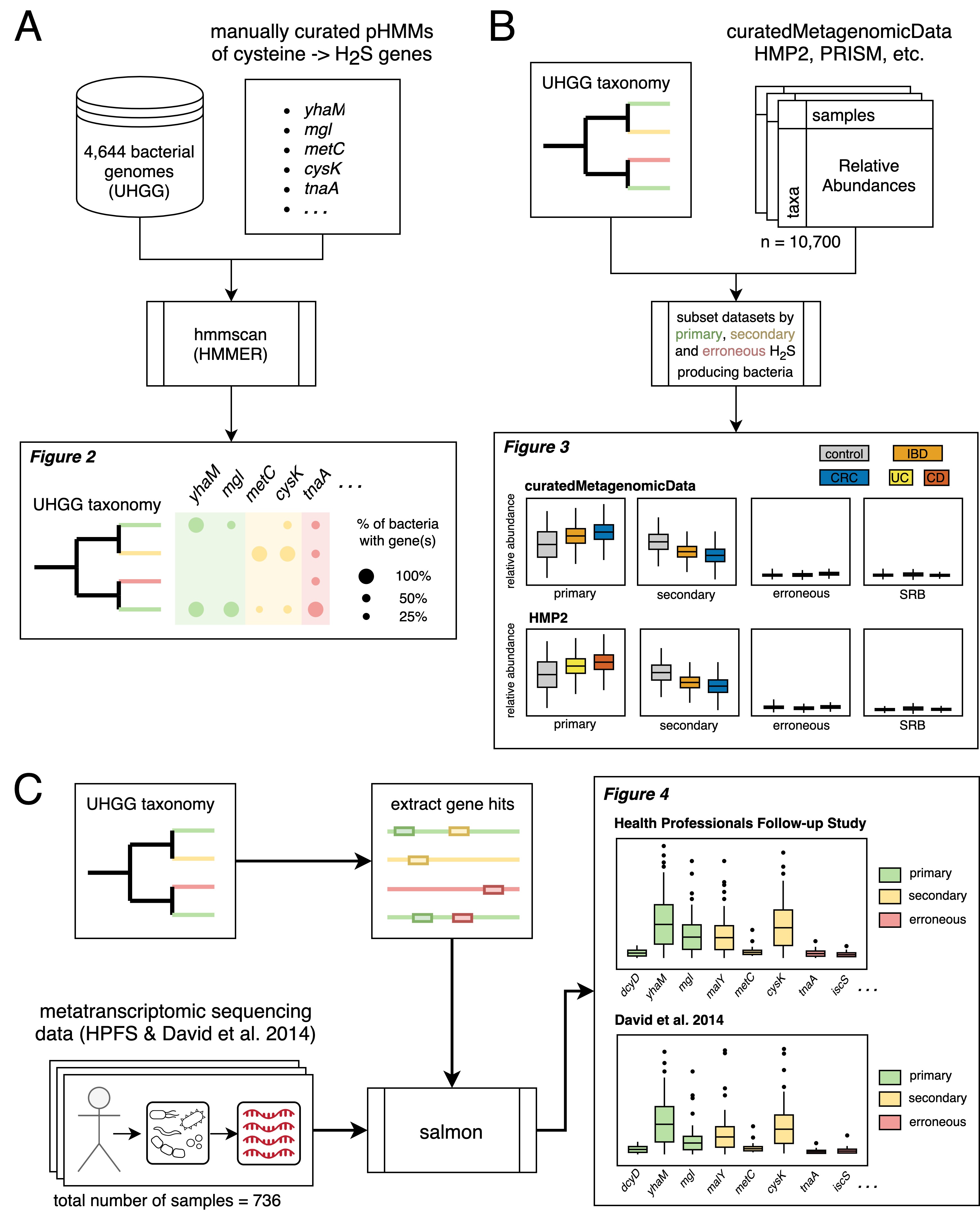

Supplement: Supplementary Figure 1 — Computational workflow. (A) Representative bacterial genomes from the United Human Gastrointestinal Genomes collection (UHGG) (Almeida et al., 2020) were downloaded and profile hidden markov models (pHMMs) representing cysteine-degrading genes were manually curated after extensive literature search (Supplementary Note 1 and Supplementary Table 1). pHMMs were searched across the UHGG database using hmmscan (HMMER) (HMMER, 2021) and gene containments are reported in Figure 2 and Supplementary Figure 2. (B) Relative abundances of putative primary, secondary and erroneous cysteine-degrading bacteria were compared across healthy, IBD and CRC individuals and reported in Figure 3. (C) Metatranscriptomic sequencing reads from HPFS (number of individuals = 308, number of samples = 677) (David et al., 2014; Abu-Ali et al., 2018) (number of individuals = 10, number of samples = 59) (David et al., 2014) were aligned and quantified against UHGG gene hits using salmon (Patro et al., 2017) and results are visualized in Figure 4 using ggplot2 (Wickham, 2011). [file Data_Sheet_2.zip › Image 1.jpg]

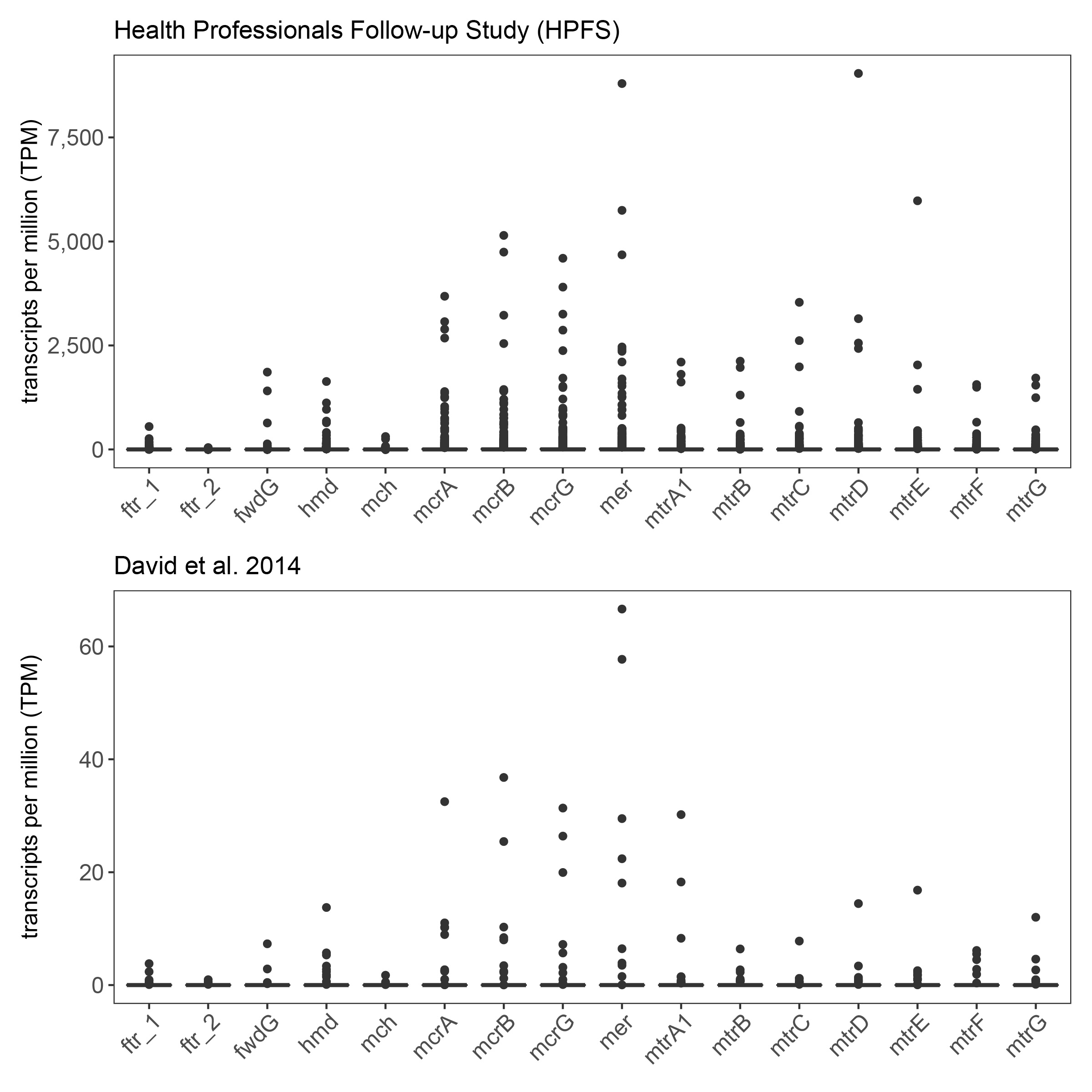

Supplement: Supplementary Figure 1 — Computational workflow. (A) Representative bacterial genomes from the United Human Gastrointestinal Genomes collection (UHGG) (Almeida et al., 2020) were downloaded and profile hidden markov models (pHMMs) representing cysteine-degrading genes were manually curated after extensive literature search (Supplementary Note 1 and Supplementary Table 1). pHMMs were searched across the UHGG database using hmmscan (HMMER) (HMMER, 2021) and gene containments are reported in Figure 2 and Supplementary Figure 2. (B) Relative abundances of putative primary, secondary and erroneous cysteine-degrading bacteria were compared across healthy, IBD and CRC individuals and reported in Figure 3. (C) Metatranscriptomic sequencing reads from HPFS (number of individuals = 308, number of samples = 677) (David et al., 2014; Abu-Ali et al., 2018) (number of individuals = 10, number of samples = 59) (David et al., 2014) were aligned and quantified against UHGG gene hits using salmon (Patro et al., 2017) and results are visualized in Figure 4 using ggplot2 (Wickham, 2011). [file Data_Sheet_2.zip › Image 3.jpg]

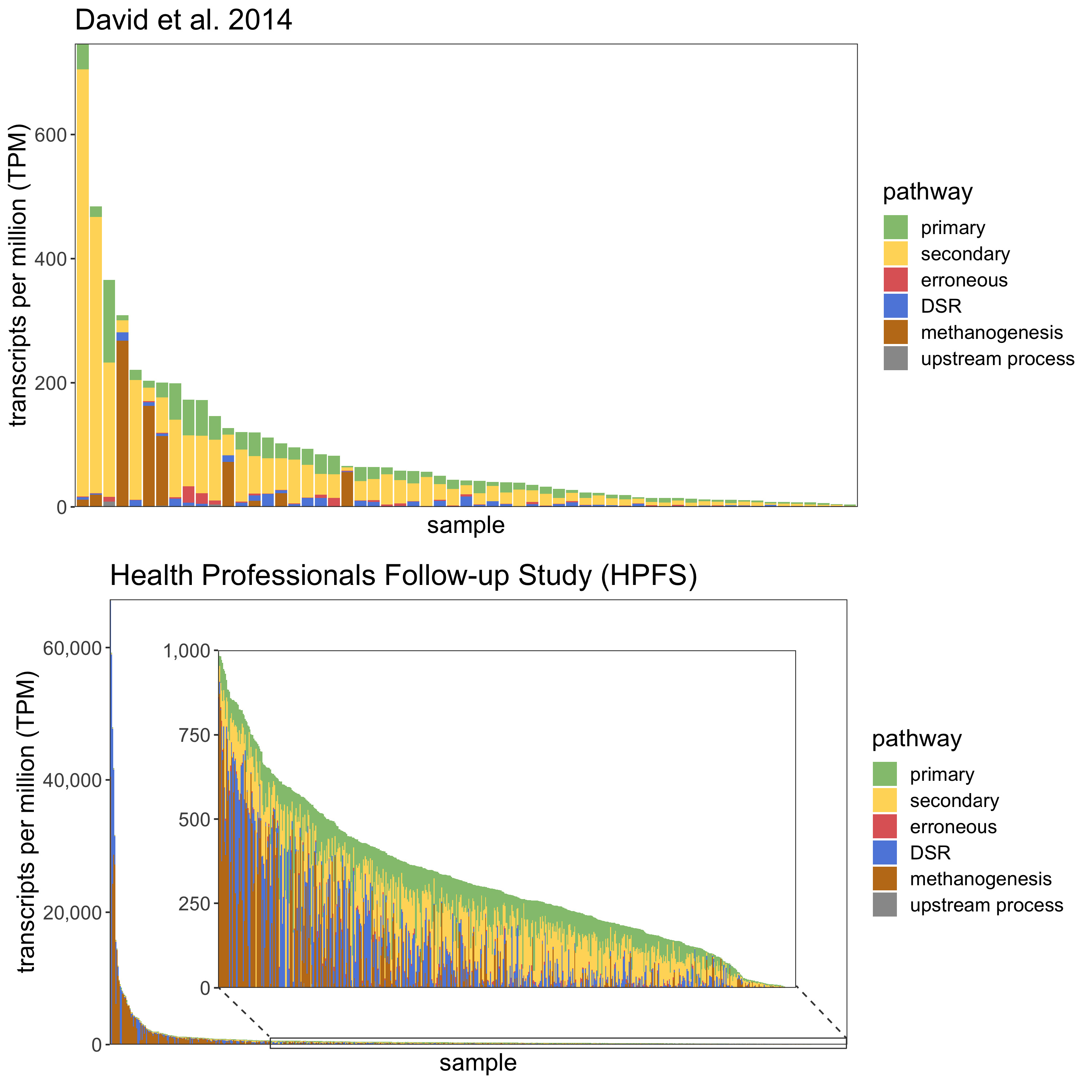

Supplement: Supplementary Figure 1 — Computational workflow. (A) Representative bacterial genomes from the United Human Gastrointestinal Genomes collection (UHGG) (Almeida et al., 2020) were downloaded and profile hidden markov models (pHMMs) representing cysteine-degrading genes were manually curated after extensive literature search (Supplementary Note 1 and Supplementary Table 1). pHMMs were searched across the UHGG database using hmmscan (HMMER) (HMMER, 2021) and gene containments are reported in Figure 2 and Supplementary Figure 2. (B) Relative abundances of putative primary, secondary and erroneous cysteine-degrading bacteria were compared across healthy, IBD and CRC individuals and reported in Figure 3. (C) Metatranscriptomic sequencing reads from HPFS (number of individuals = 308, number of samples = 677) (David et al., 2014; Abu-Ali et al., 2018) (number of individuals = 10, number of samples = 59) (David et al., 2014) were aligned and quantified against UHGG gene hits using salmon (Patro et al., 2017) and results are visualized in Figure 4 using ggplot2 (Wickham, 2011). [file Data_Sheet_2.zip › Image 4.jpg]
