# Supplementary material for: The Capacity to Produce Hydrogen Sulfide (H2S) via Cysteine Degradation Is Ubiquitous in the Human Gut Microbiome
Source: Front Microbiol. 2021 Oct 20;12:705583. doi: 10.3389/fmicb.2021.705583 (PMC8564485; doi:10.3389/fmicb.2021.705583)
Supplement: Supplementary file 2 [file Data_Sheet_1.DOCX]

**Characterization of H_2_S producing genes included in the search space**

For each gene considered, we stated 1. the main species the gene is found in (limited to gut inhabiting bacteria), 2. whether the downstream enzyme is dependent on pyridoxal 5’-phosphate (PLP) binding, and 3. the primary function of the gene with references.

- **Green** highlighted genes indicate that H_2_S output is central to the **primary** function of the gene and/or these genes are upregulated in the presence of cysteine.
- **Yellow** highlighted genes indicate that H_2_S output is a **secondary** function of the gene and/or is closely related to cysteine degradation. Most of these genes, apart from *malY*, are involved in some component of the transulfuration pathway and additionally exhibit cysteine desulfurase activity.
- **Red** highlighted genes indicate that the gene has been shown to produce H_2_S from cysteine, but the primary function of the gene is unrelated to H_2_S production and/or the gene does appear to be upregulated in the presence of cysteine. We refer to these genes as **erroneous** H2S producing genes.

| ***gene abbreviation*** | **Full gene name** |
| --- | --- |

| ***dcyD*** | **Cysteine desulfhydrase** |
| --- | --- |

- Main species:
  - *Klebsiella pneumoniae (many strains)*
  - *Escherichia coli (K-12)*
- UniProt Entry: <https://www.uniprot.org/uniprot/A6TB69>
- PLP-dependent: **YES**
- Primary function: catalyzes the the beta elimination of D-cysteine and other D-cysteine derivatives. Potentially useful for detoxification of D-cysteine. (1,2)

| ***yhaOM* operon** | **Unnamed proteins** |
| --- | --- |

- Main species:
  - *Escherichia coli (K-12)*
- PLP-dependent: **NO**
- Primary function: yhaO and yhaM are genes in the yhaOM operon, regulated by decR in E. coli (3). *yhaO* is likely a serine/cysteine transporter protein and *yhaM* has cysteine desulfurase activity (3). Of all the cysteine desulfurase active proteins in *E. coli, yhaM* has been proposed to be the primary cysteine desulfurase protein (4), as many of the other genes identified by Awona et al. (5,6) have other primary functions such as Fe-S complex formation in the case of *iscS*.

| ***mgl*** | **Methionine gamma-lyase** |
| --- | --- |

- Main species:
  - *Fusobacterium nucleatum*
  - *Porphyromonas gingivalis*
  - *Treponema denticola*
- PLP-dependent: **YES**
- Primary function: The above species are the most frequently encountered species in the human oral microbiome, and have been implicated in periodontitis (7). Due to the mucosal-degrading activity of methanethiol (CH_3_SH) and hydrogen sulfide (H_2_S), it is believed by some that *mgl* is actively expressed in the presence of methionine and cysteine and acts as a pathogenicity factor (8).

| ***aspC* + *sseA*** | **Cysteine aminotransferase + 3-mercaptopyruvate sulfurtransferase** |
| --- | --- |

- Main species:
  - *Escherichia coli (K-12)*
  - *aspC* found in *E. coli K-12*
  - Many other species have *sseA* predicted based on homology including: *Yersinia spp., Salmonella spp., Brucella spp., Clostridium spp.* and *Klebsiella spp. (9)*
- PLP-dependent: *aspC* – **YES**; *sseA* – **NO**
- Primary function: Proposed by Shatalin et al. (9), H_2_S production by *sseA* leads to decreased antibiotic sensitivity. However, it appears that other modes of H_2_S production are more prominent in anerobic environments (10). This mechanism is more complicated, because *sseA* acts on 3-mercaptopyruvate, which is produced from cysteine by a cysteine aminotransferase, such as *aspC* (11).

| ***metC*** | **Cystathionine beta-lyase** |
| --- | --- |

- Main species:
  - *Escherichia coli (K-12)*
  - *Bacillus subtilis (168)*
- PLP-dependent: **YES**
- Primary function: Involved in the transulfuration pathway (cysteine to methionine interconversion). Specifically, *metC* reversibly converts cystathionine to homocysteine (5,12,13). Any cysteine desulfhydrase activity resulting in H2S production seems to be secondary to the primary function of cystathionine to homocysteine interconversion (3).

| ***malY*** | **Cystathionine beta-lyase like; repressor of maltose regulon** |
| --- | --- |

- Main species:
  - *Escherichia coli (K-12)*
- PLP-dependent: **YES**
- Primary function: *malY* is one of the few instances of a truly bi-functional protein on this list, serving as both a cystathionine beta-lyase (*metC*-like) and as a repressor of the maltose regulon responsible for the uptake and metabolism of maltose and maltodextrins (14,15). *malY* can recapitulate cystathionine beta-lyase activity in *metC* mutant strains of *E. coli*.

| ***cysK*** | **Cysteine synthase A** |
| --- | --- |

- Main species:
  - *Escherichia coli (K-12)*
  - *Bacillus subtilis (168)*
- PLP-dependent: **YES**
- Primary function: converts O-acetyl-serine + H_2_S -> acetate + cysteine (13), however, the pathway appears to be reversible, resulting in H2S production from cysteine (3,6). Additionally, appears to be crucial in contact dependent growth inhibition of competing bacteria (16).

| ***cysM*** | **Cysteine synthase B** |
| --- | --- |

- Main species:
  - *Escherichia coli (K-12)*
  - *Bacillus subtilis (168)*
- PLP-dependent: **YES**
- Primary function: converts O-acetyl-serine + H_2_S -> acetate + cysteine (13), however, the pathway appears to be reversible, resulting in H2S production from cysteine (3,6).

| ***mccB*** | **Cystathionine gamma-lyase** |
| --- | --- |

- Main species:
  - *Bacillus subtilis (168)*
- PLP-dependent: **YES**
- Primary function: Catalyzes the conversion of cystathionine -> cysteine. Can also catalyze a similar gamma-lyase reaction converting homocysteine + H_2_O -> H_2_S + 2-oxobutanoate + ammonia + pyruvate (13). Due to the potentially bifunctionality of this gene, it may be upgraded from erroneous to secondary production of H_2_S.

| ***tnaA*** | **Tryptophanase** |
| --- | --- |

- Main species:
  - *Escherichia coli (K-12)*
  - *Proteus vulgaris*
  - *Shigella flexneri*
  - *Klebsiella aerogenes*
- PLP-dependent: **YES**
- Primary function: Catalyzes the degradation of H_2_O + tryptophan -> indole + ammonia + pyruvate (17). *tnaA* has been shown to perform cysteine dehydratase activity under certain circumstances (5), but the upregulation of *tnaA* expression and activity could not be replicated in future studies (4). It is possible that the primary function of *tnaA* is tryptophan degradation and any cysteine desulhydrase activity is erroneous.

| ***iscS*** | **Cysteine desulfurase** |
| --- | --- |

- Main species:
  - *Escherichia coli (K-12)*
  - (very common gene in all of life)
- PLP-dependent: **YES**
- Primary function: *iscS* is potentially the most clear-cut case of erroneous H2S production by gut bacteria. *iscS* is a master enzyme that transfers sulfur from cysteine to aid in Fe-S cluster assembly, a key structure in the formation of many proteins (18) (and many other references). However, it should be noted that the production of H_2_S mediated by *iscS* in E. coli can still prove to be quite substantial in anaerobic and high cysteine conditions (10).

| ***mccA*** | **O-acetylserine dependent cystathionine beta-synthase** |
| --- | --- |

- Main species:
  - *Bacillus subtilis (168)*
  - *Staphylococcus aureus*
- PLP-dependent: **YES**
- Primary function: Converts O-acetylserine and homocysteine to cystathionine (13). Given the primary function of the enzyme, it appears that any cysteine desulfurase activity is either secondary or erroneous.

References

1. Nagasawa T, Ishii T, Kumagai H, Yamada H. D-Cysteine desulfhydrase of Escherichia coli. Purification and characterization. Eur J Biochem. 1985 Dec 16;153(3):541–51.

2. Soutourina J, Blanquet S, Plateau P. Role of d-Cysteine Desulfhydrase in the Adaptation of Escherichia coli to d-Cysteine. J Biol Chem. 2001 Nov 2;276(44):40864–72.

3. Shimada T, Tanaka K, Ishihama A. Transcription factor DecR (YbaO) controls detoxification of L-cysteine in Escherichia coli. Microbiology. 2016 Sep 1;162(9):1698–707.

4. Nonaka G, Takumi K. Cysteine degradation gene yhaM, encoding cysteine desulfidase, serves as a genetic engineering target to improve cysteine production in Escherichia coli. AMB Express. 2017 May 10;7(1):90.

5. Awano N, Wada M, Kohdoh A, Oikawa T, Takagi H, Nakamori S. Effect of cysteine desulfhydrase gene disruption on L-cysteine overproduction in Escherichia coli. Appl Microbiol Biotechnol. 2003 Aug;62(2–3):239–43.

6. Awano N, Wada M, Mori H, Nakamori S, Takagi H. Identification and Functional Analysis of Escherichia coli Cysteine Desulfhydrases. Appl Environ Microbiol. 2005 Jul 1;71(7):4149–52.

7. Suwabe K, Yoshida Y, Nagano K, Yoshimura F. Identification of an l-methionine γ-lyase involved in the production of hydrogen sulfide from l-cysteine in Fusobacterium nucleatum subsp. nucleatum ATCC 25586. Microbiology,. 2011;157(10):2992–3000.

8. Ng W, Tonzetich J. Effect of Hydrogen Sulfide and Methyl Mercaptan on the Permeability of Oral Mucosa. J Dent Res. 1984 Jul 1;63(7):994–7.

9. Shatalin K, Shatalina E, Mironov A, Nudler E. H2S: A Universal Defense Against Antibiotics in Bacteria. Science. 2011 Nov 18;334(6058):986–90.

10. Wang J, Guo X, Li H, Qi H, Qian J, Yan S, et al. Hydrogen Sulfide From Cysteine Desulfurase, Not 3-Mercaptopyruvate Sulfurtransferase, Contributes to Sustaining Cell Growth and Bioenergetics in E. coli Under Anaerobic Conditions. Front Microbiol [Internet]. 2019 [cited 2021 Jan 19];10. Available from: http://www.frontiersin.org/articles/10.3389/fmicb.2019.02357/full

11. Andreeßen C, Gerlt V, Steinbüchel A. Conversion of cysteine to 3‐mercaptopyruvic acid by bacterial aminotransferases. Enzyme Microb Technol. 2017 Apr 1;99:38–48.

12. Auger S, Gomez MP, Danchin A, Martin-Verstraete I. The PatB protein of Bacillus subtilis is a C-S-lyase. Biochimie. 2005 Feb 1;87(2):231–8.

13. Hullo M-F, Auger S, Soutourina O, Barzu O, Yvon M, Danchin A, et al. Conversion of methionine to cysteine in Bacillus subtilis and its regulation. J Bacteriol. 2007 Jan;189(1):187–97.

14. Zdych E, Peist R, Reidl J, Boos W. MalY of Escherichia coli is an enzyme with the activity of a beta C-S lyase (cystathionase). J Bacteriol. 1995 Sep;177(17):5035–9.

15. Clausen T, Schlegel A, Peist R, Schneider E, Steegborn C, Chang Y-S, et al. X-ray structure of MalY from Escherichia coli: a pyridoxal 5′-phosphate-dependent enzyme acting as a modulator in mal gene expression. EMBO J. 2000 Mar 1;19(5):831–42.

16. Johnson PM, Beck CM, Morse RP, Garza-Sánchez F, Low DA, Hayes CS, et al. Unraveling the essential role of CysK in CDI toxin activation. Proc Natl Acad Sci U S A. 2016 Aug 30;113(35):9792–7.

17. DeMoss R. D., Moser K. Tryptophanase in Diverse Bacterial Species. J Bacteriol. 1969 Apr 1;98(1):167–71.

18. Schwartz CJ, Djaman O, Imlay JA, Kiley PJ. The cysteine desulfurase, IscS, has a major role in in vivo Fe-S cluster formation in Escherichia coli. Proc Natl Acad Sci. 2000 Aug 1;97(16):9009–14.
